# Supplementary material for: Temperature-dependent kinetic pathways of heterogeneous ice nucleation competing between classical and non-classical nucleation
Source: Nat Commun. 2021 Aug 16;12:4954. doi: 10.1038/s41467-021-25267-2 (PMC8367957; doi:10.1038/s41467-021-25267-2)
Supplement: Supplementary file 1 — Supplementary information [file 41467_2021_25267_MOESM1_ESM.pdf]

SUPPLEMENTARY INFORMATION

Temperature-dependent Kinetic Pathways of Heterogeneous Ice Nucleation Competing between Classical and Non-classical Nucleation

Chu Li[1], Zhuo Liu[1,2], Eshani C. Goonetilleke[1], Xuhui Huang[*1]

[1]*Department of Chemistry, Center of Systems Biology and Human Health, State Key Laboratory of Molecular Neuroscience, The Hong Kong University of Science and Technology, Kowloon, Hong Kong*

[2]*Institute for Advanced Study, The Hong Kong University of Science and Technology, Clear Water Bay, Kowloon, Hong Kong*

* To whom correspondence should be addressed. Email: xuhuihuang@ust.hk

Supplementary Figures

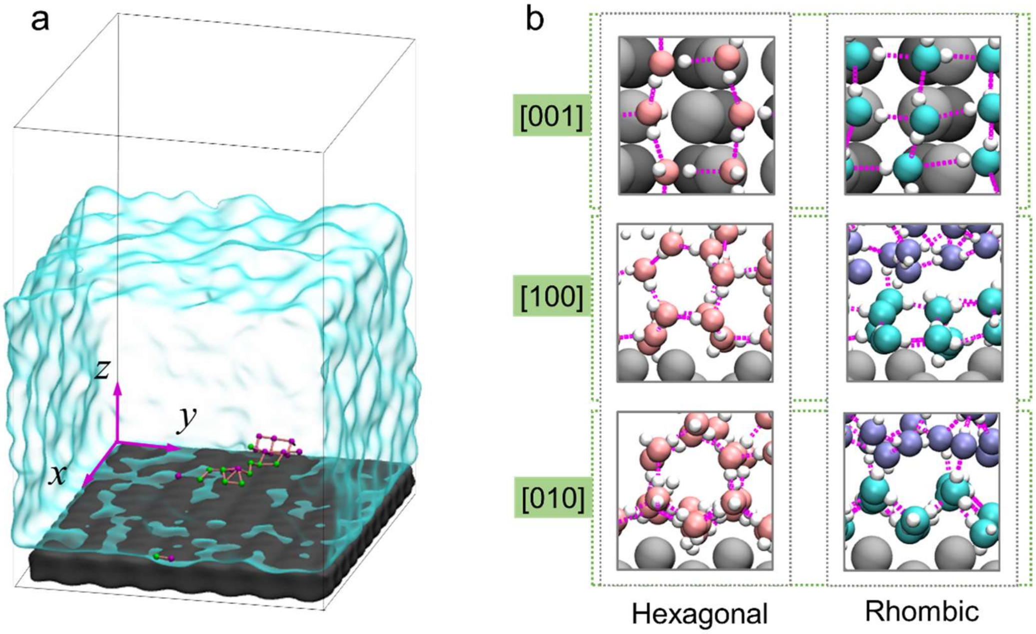

Supplementary Figure 1. (a) Molecular dynamics simulation system. A typical simulation system consists of a slab of water (transparent cyan) and a surface (black). (b) Typical hexagonal ice (pink) and rhombic ice (cyan) close to the surface from different angles. Only one layer of rhombic ice can form due to the lack of dangling H-bonds, thus water (purple) above the rhombic ice remains in the liquid state. The H-bonds are represented by the magenta dashed lines.

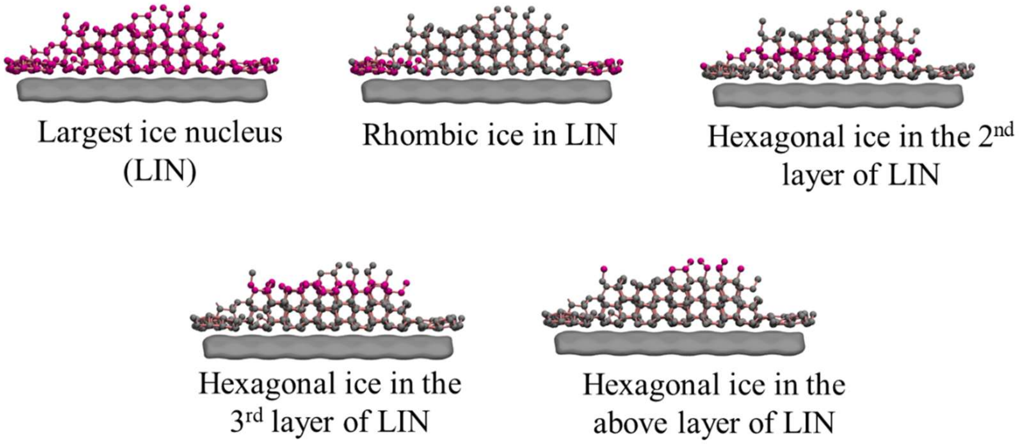

Supplementary Figure 2. Illustration of the five selected representative collective variables (CVs). The five CVs represent the numbers of ice molecules in the corresponding configurations as colored in purple: the largest ice nucleus, rhombic ice in the largest ice nucleus, and hexagonal ice in the 2nd, 3rd, and upper layers of the largest ice nucleus, respectively.

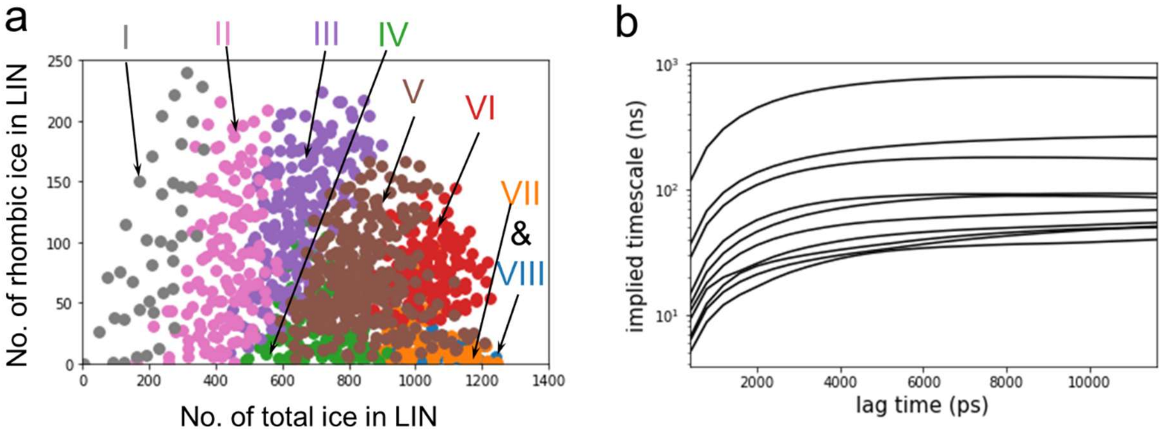

Supplementary Figure 3. Markov State Model (MSM) at *T*=230 K. (a) Lumping microstates into eight macro-states using PCCA+. Each dot projected on two major CVs represents a microstate, and each color indicates a macro-state. (b) The implied timescales plotted against lag time for the 1000-microstate MSMs. The curves level off at about 5 ns, suggesting that the MSMs reach Markovian at lag time greater than or equal to ~5ns.

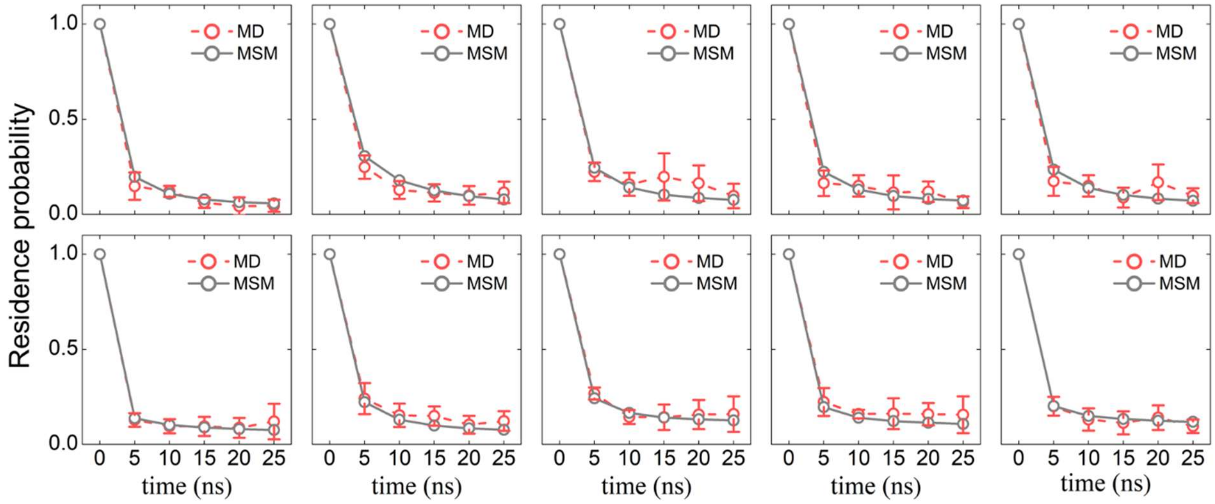

Supplementary Figure 4. Validation of Markov State Models (MSMs) by comparing the residence probabilities of the system remaining in the same microstates for the ten most populated microstates obtained from MSMs. This is done by propagating the transition matrix and the original molecular dynamic trajectories, *T*=230 K. The error bars represent the standard errors with the original molecular dynamic trajectories sampled by bootstrapping.

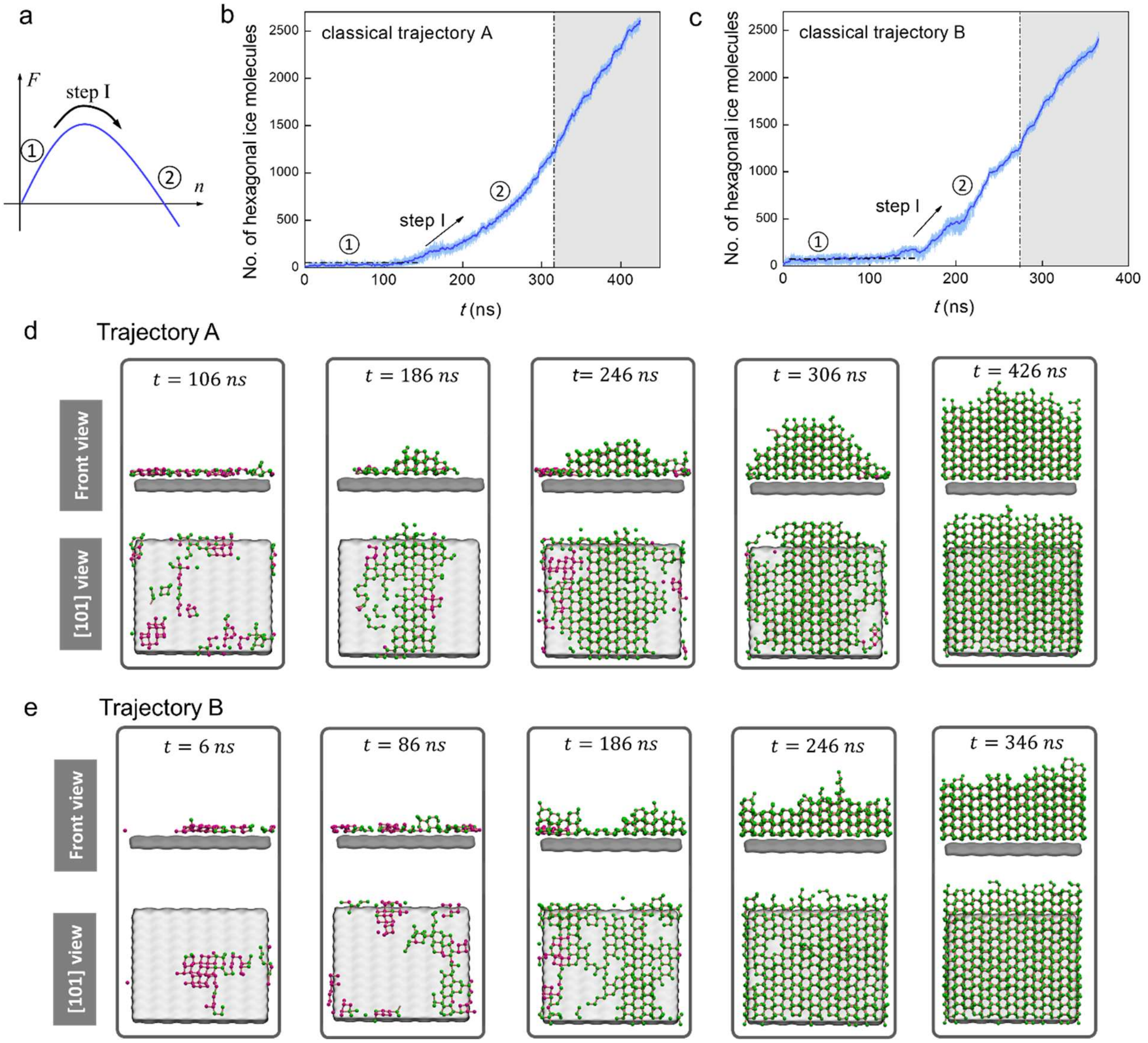

Supplementary Figure 5. Typical molecular dynamics trajectories of the classical one-step nucleation pathway at T =230 K. (a). Schematic of the free energy profile for the classical ice nucleation pathway. (b)-(c). Evolutions of the number of hexagonal ice molecules in the largest ice nucleus, exhibiting one activation process. The gray regions are extended simulations showing that ice grows continuously after entering the growth stage. (d)-(e). Typical configurations of the largest ice nucleus for the trajectories in (b) and (c), respectively. The purple and green spheres denote the rhombic and hexagonal ice, respectively. The gray surfaces represent the substrate.

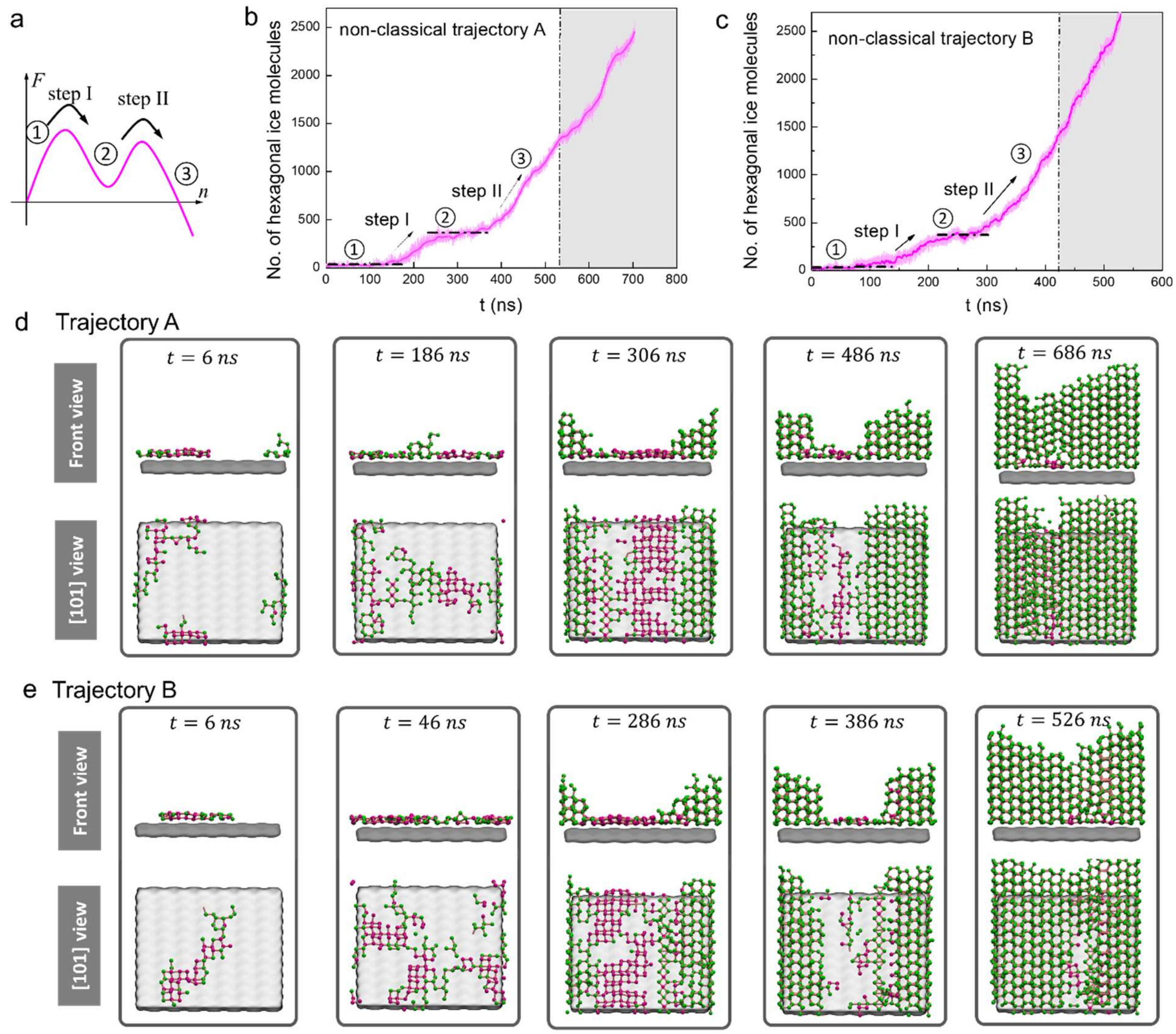

Supplementary Figure 6. Typical molecular dynamics trajectories of the non-classical two-step nucleation pathway at T =230 K. (a). Schematic of the free energy profile for the non-classical two-step ice nucleation pathway. (b)-(c). Evolutions of the number of hexagonal ice molecules in the largest ice nucleus, exhibiting two activation processes. The gray regions are extended simulations showing that ice grows continuously after entering the growth stage. (d)-(e). Typical configurations of the largest ice nucleus for the trajectories in (b) and (c), respectively. The purple and green spheres denote rhombic and hexagonal ice, respectively. The gray surfaces represent the substrate.

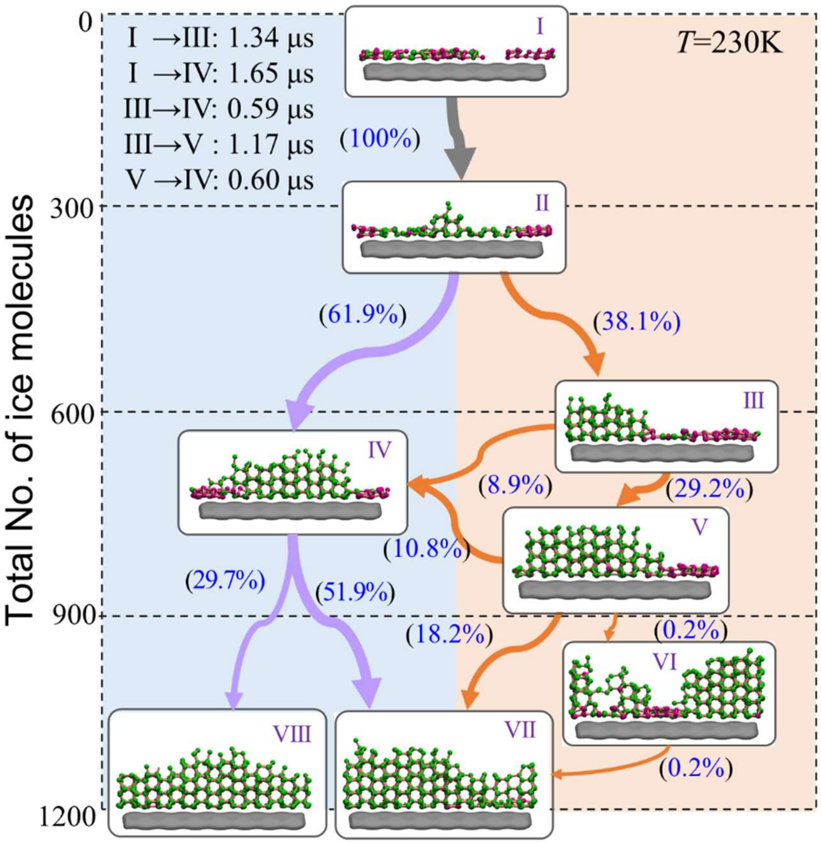

Supplementary Figure 7. Percentages of complete flux for heterogeneous ice nucleation at *T*=230 K. The numbers on the left give the total number of ice molecules. The pathways following the purple and yellow arrows depict the classical one-step and nonclassical two-step nucleation pathways, respectively. The numbers in brackets next to each arrow represent the flux as a percentage (shown in blue) for each transition between each pair of states. The mean first passage time of some transitions are listed in black in the top-left corner. After macro-states IV or V, the system enters the stage of ice growth.

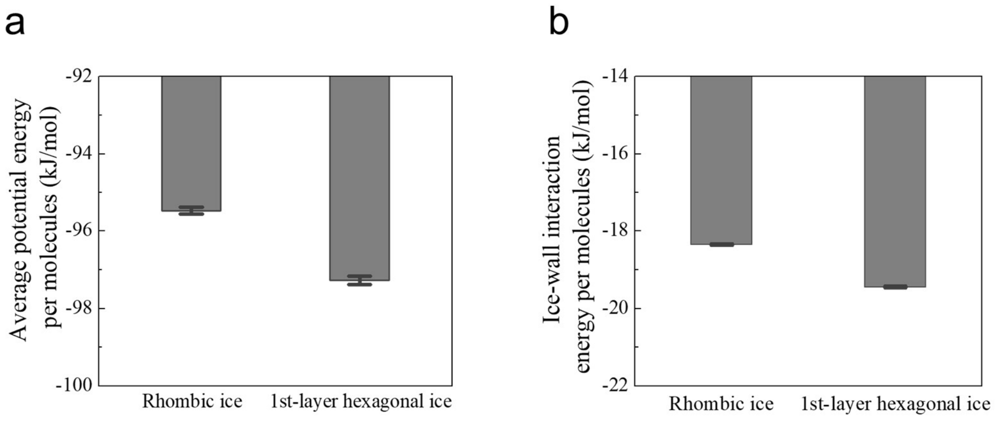

Supplementary Figure 8. Average potential energy per molecule for the 1st layer of hexagonal ice and rhombic ice in TS II at *T*=230 K. (a). Total potential energy for the 1st layer of hexagonal ice and rhombic ice. (b). Ice-surface interaction energy for the 1st layer of hexagonal ice and rhombic ice. The error bars represent the standard errors with samples obtained by bootstrapping.

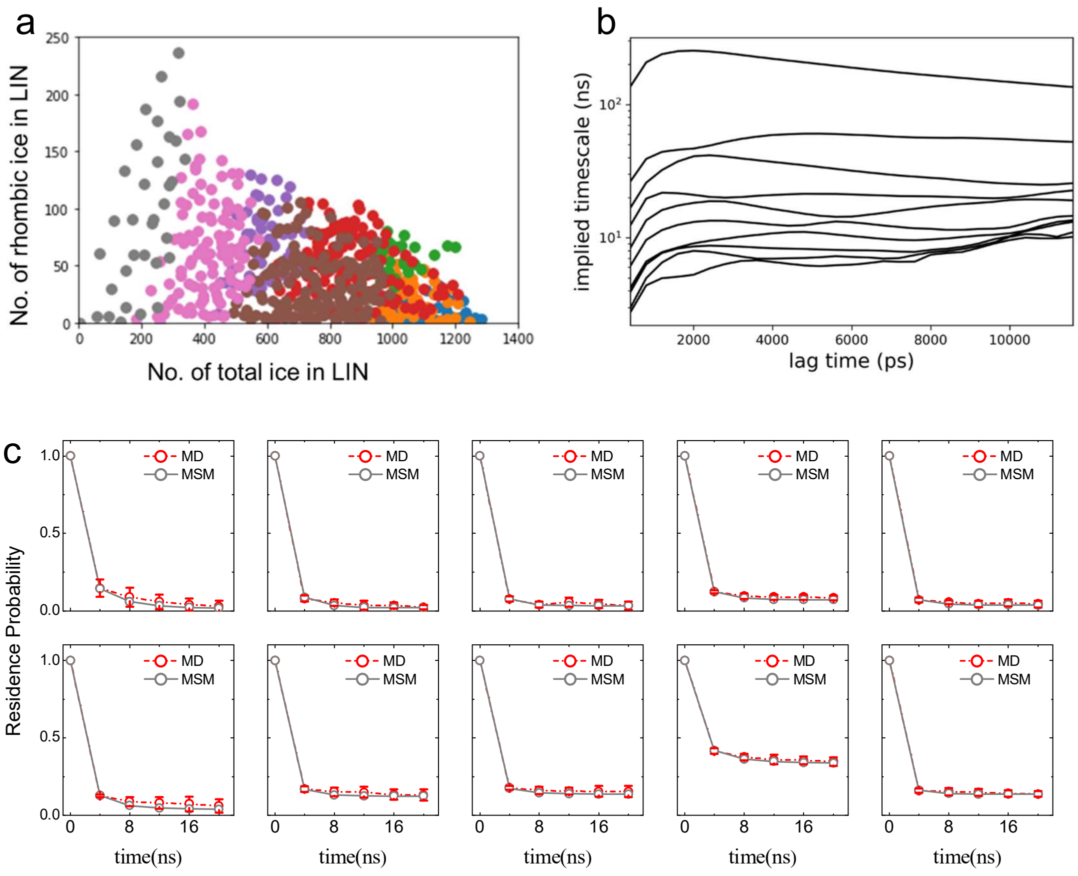

Supplementary Figure 9. Construction and validation of the Markov State Model (MSM) at *T*=240 K. The microstates are generated by projecting the molecular dynamic (MD) trajectories onto the original microstates for *T*=230 K. Using the same *k*-centers-generated microstates allows us to directly compare the flux along different pathways. (a) The microstates taken by the trajectories at *T*=240 K. Each dot projected on two major features represents a microstate and each color indicates a macro-state. (b) Implied timescales plotted against lag time for the projected microstate MSM. The MSMs reach Markovian at a lag time greater than or equal to ~4ns. (c) Validation of the MSM by comparing the residence probabilities of the system remaining in the same microstates for the ten most populated microstates obtained from MSM by propagating the transition matrix and the original MD trajectories. The error bars represent the standard errors with the original molecular dynamic trajectories sampled by bootstrapping.

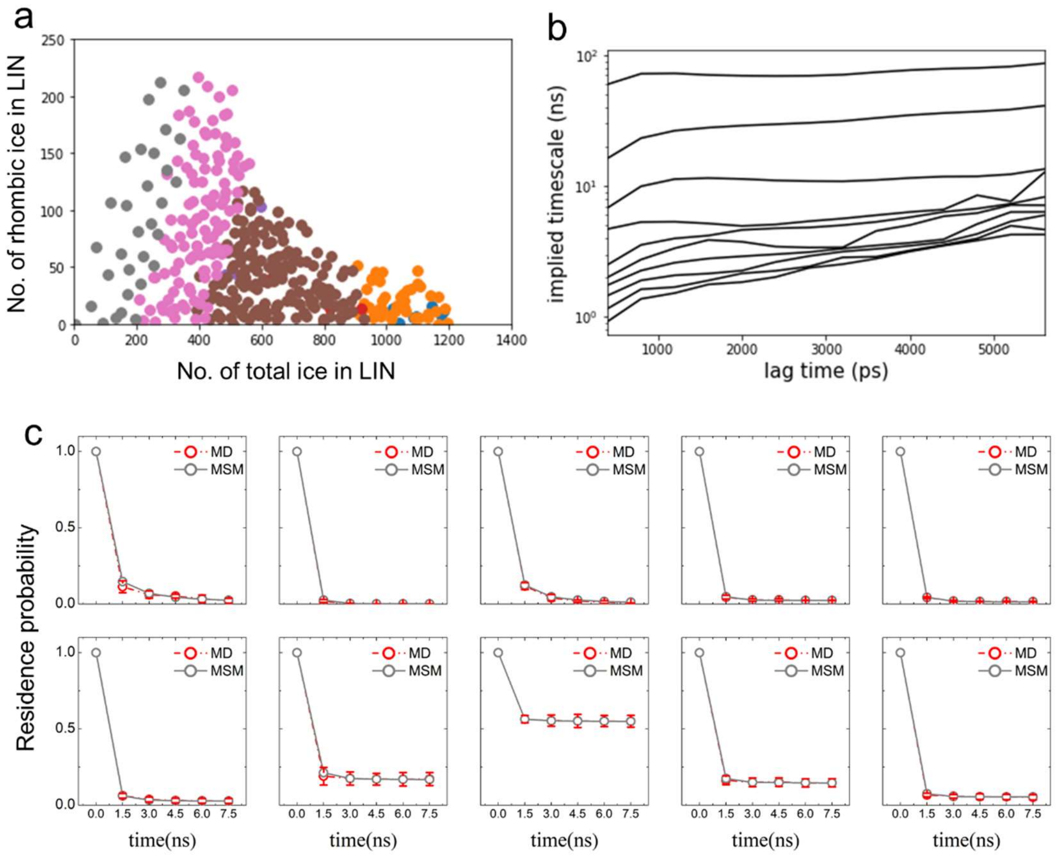

Supplementary Figure 10. Construction and validation of the Markov State Model (MSM) at T=250 K. (a) The microstates taken by the trajectories at T=250 K. The microstates are generated by projecting the molecular dynamic (MD) trajectories onto the original microstates for T=230 K. Each dot projected on two major features represents a microstate and each color indicates a macro-state. (b) Implied timescales plotted against lag time for the projected-microstates MSM. The MSMs reach Markovian at a lag time greater than or equal to ~1.5ns. (c) Validation of the MSM by comparing the residence probabilities of the system remaining in the same microstates for the ten most populated microstates obtained from MSM by propagating the transition matrix and the original MD trajectories. The error bars represent the standard errors with the original molecular dynamic trajectories sampled by bootstrapping.

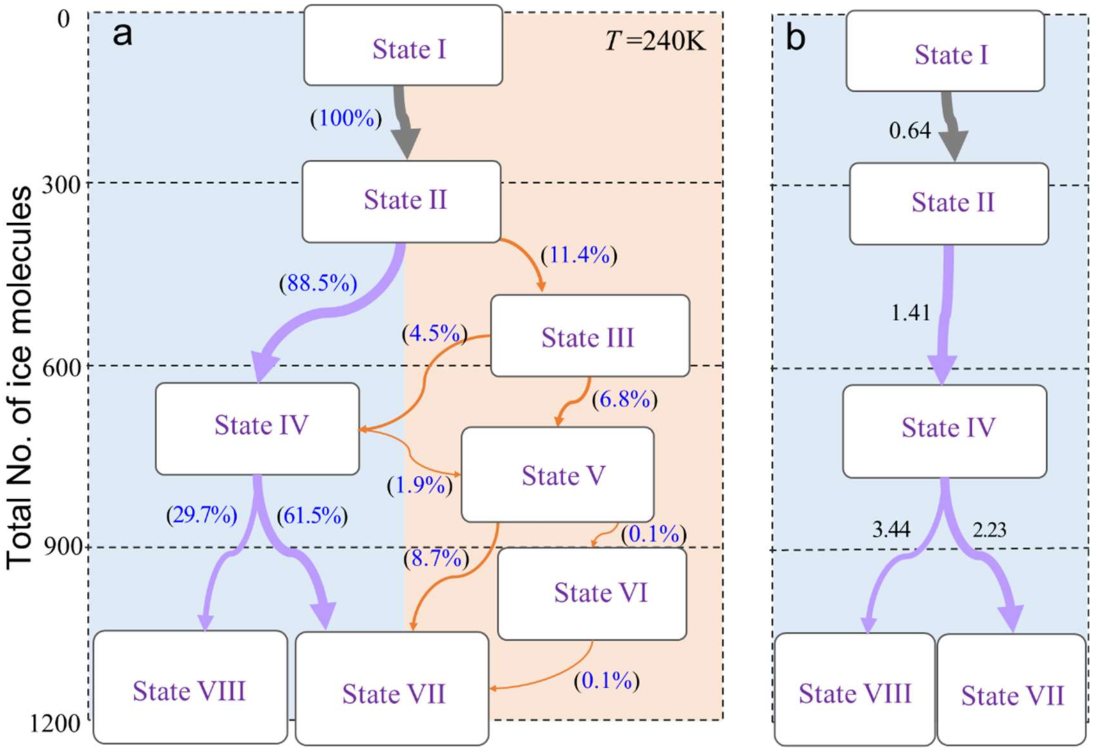

Supplementary Figure 11. Kinetics of HIN at T=240 K. (a) Percentages of complete flux for HIN. The pathways following the purple and yellow arrows depict the classical one-step and nonclassical two-step nucleation pathways, respectively. The number in brackets next to each arrow (shown in blue) represents the flux as a percentage for each transition between each pair of states. (b) The mean first passage time (MFPT) (shown in black, in unit of μs) for the classical pathway between each pair of macro-states.

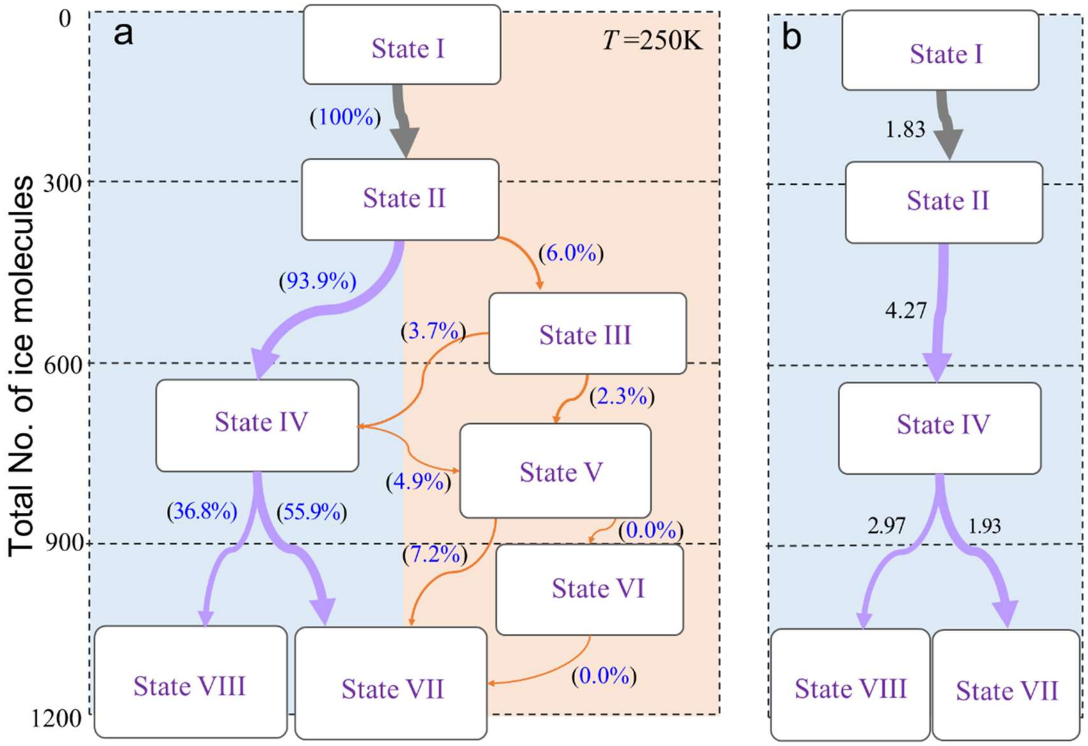

Supplementary Figure 12. Kinetics of HIN at T=250 K. (a) Percentages of complete flux for HIN. The pathways following the purple and yellow arrows depict the classical one-step and nonclassical two-step nucleation pathways, respectively. The number in brackets next to each arrow (shown in blue) represents the flux as a percentage for each transition between each pair of states. (b) The mean first passage time (MFPT) (shown in black, in unit of μs) for the classical pathway between each pair of macro-states.

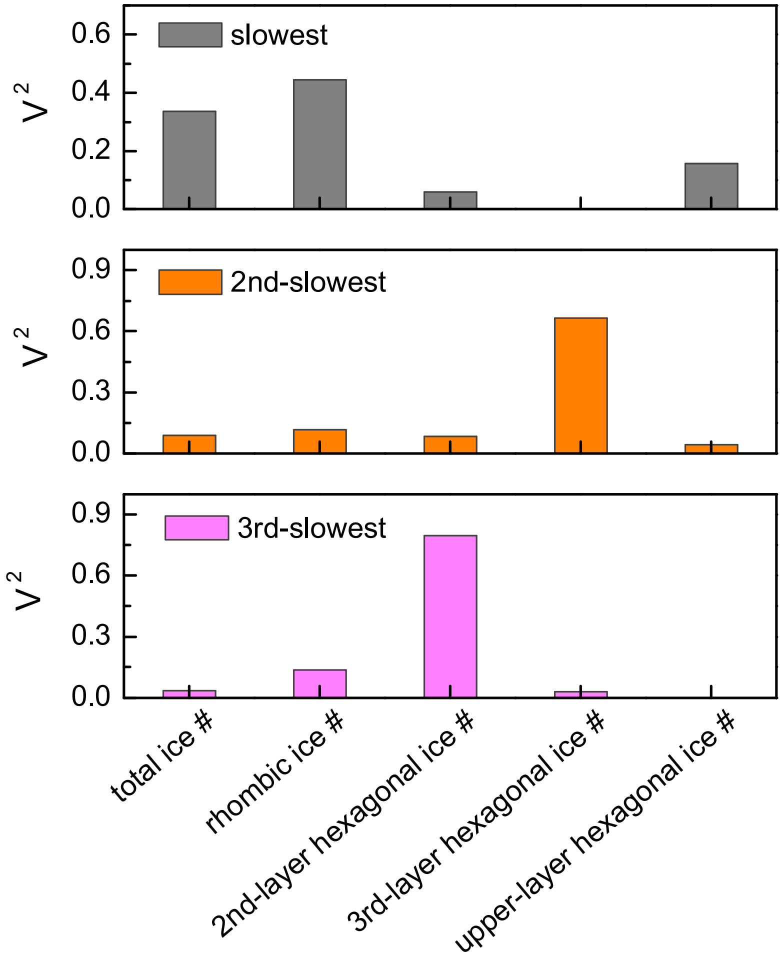

Supplementary Figure 13. Contributions of the CVs to the timescales. To evaluate the contributions, we took the three leading eigenvectors that correspond to the three slowest timescales, and then normalized them based on the squared sum of their components. The normalized square of each component in each eigenvector, V^2, is plotted as the relative contribution. A higher V^2 value indicates a stronger contribution to the slowest timescales. The leading eigenvectors were obtained from the time-lagged correlation matrix from the Spectral-oASIS analysis with the five chosen CVs as shown in Fig. 1c in the main text.

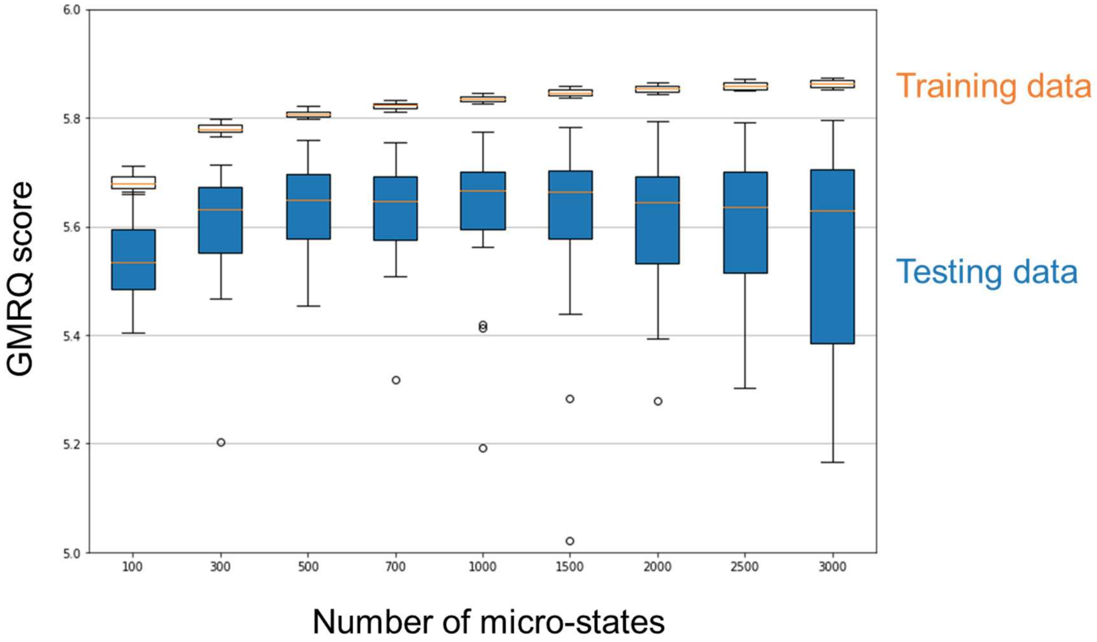

Supplementary Figure 14. Variational cross-validation for the selection of the number of microstates with a generalized matrix Rayleigh quotient (GMRQ). Boxes indicate median and quartiles, while whiskers follow the default definition in matplotlib package. The scattering dots indicate outliers. 1000 was chosen as the optimal number of microstates, achieving the highest GMRQ score for the testing data.

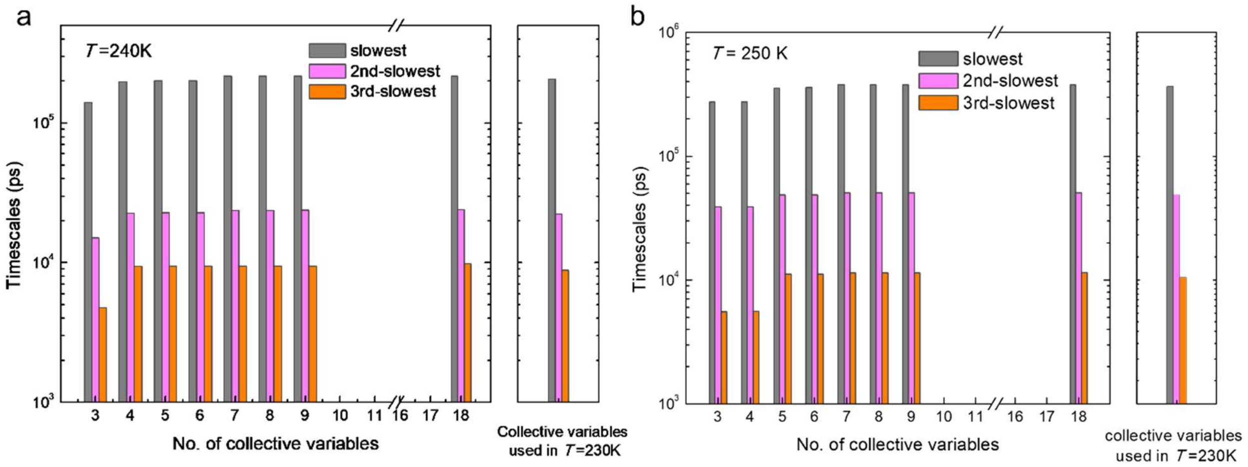

Supplementary Figure 15. Timescales for the dynamics of the system with various numbers of CVs at T =240 K (a) and 250 K (b), respectively. The left panels present timescales analyzed by spectral-oASIS and the right panels are the timescales when using the CVs obtained at T =230 K.

Supplementary Table

Supplementary Table 1. List of potential collective variables (CVs) for Nyström-oASIS analysis to choose the representative CVs.

No.	candidate	No.	candidate
0	Size of largest hexagonal ice (Ih) nucleus	9	No. of ice in the upper layer of LIN
1	Size of largest rhombic ice (Ir) nucleus	10	No. of Ih in the 1st layer of LIN
2	Size of largest ice nucleus (LIN)	11	No. of Ih in the 2nd layer of LIN
3	No. of Ir in LIN	12	No. of Ih in the 3rd layer of LIN
4	No. of Ih in LIN	13	No. of Ih in the upper layer of LIN
5	Spherical parameter as defined in ref.[30]	14	No. of Ir in the 1st layer of LIN
6	No. of ice in the 1st layer of LIN	15	No. of Ir in the 2nd layer of LIN
7	No. of ice in the 2nd layer of LIN	16	No. of Ir in the 3rd layer of LIN
8	No. of ice in the 3rd layer of LIN	17	No. of Ir in the upper layer of LIN

Supplementary Methods

Supplementary Method 1. System setup and all-atom MD simulations

The all-atom *NVT* molecular dynamics (MD) simulations[1] were performed using LAMMPS packages[2] with a time step of 2 fs. The system consisted of a slab of water (4940 water molecules) sitting on top of a wurtzite-structured surface in a periodic box with dimensions: x=5.43 nm, y= 5.89 nm, and z= 7.0 nm; and periodic boundary conditions (PBC) were employed in the *xyz* directions, respectively. To minimize the undesired water-surface interactions caused by the PBC, a void space (around 3nm) was maintained above the water in the z-direction. Additionally, the surface atoms were fixed in the wurtzite structure[3] with unit cell parameters of a=4.519 Å and c=7.357 Å. To simplify the modelled surface, the charge effect of the surface atoms was not considered. Furthermore, the TIP4P/Ice[4] water model was applied to simulate the water molecules, whose bonds and angles were maintained by the SHAKE algorithm[5,6]. The water-surface interactions were modelled by the 12-6 Lennard-Jones (LJ) potential[7], which was cut off at 1.0 nm with interaction strength $\varepsilon_{\mathrm{ws}}$=4.98829 kJ mol^{-1} and σ=2.9034 Å. The water-surface interaction strength is close to, but a bit less than, that of gold-water interactions[8], i.e., $\varepsilon_{\mathrm{O-A}}$ =5.2362576 kJ mol^{-1}. The Columbic interactions among the water molecules were cut off at 0.85 nm, whose long-range interactions were considered by the Particle-Particle Particle-Mesh (PPPM)[9].

The system was initially heated up to 300 K for 2ns before being cooled down to a target temperature*, i.e.*, 230 K and 240 K. The temperature was controlled by the Nosé-Hoover thermostat[10–13], and the data was collected for production after relaxation. The hexagonal ice nucleates and then grows mainly on its first prism plane. To account for the possibility of both the one-step and two-step pathways, unbiased MD simulations were run in parallel with different initial velocities at T=230 K and 240 K. The simulations were terminated once the total number of ice molecules (n_{ice}) reached about 1400, or the simulation time exceeded 1000 ns. In total, the simulation length was about 15 μs for each temperature.

At T=250 K, two rounds of seeded unbiased MD simulations were performed. In the first round, 200 initial seeds were randomly chosen as the starting structures from the cluster centers of the most populated microstates at T=240 K. Unbiased MD simulations were then carried out with different initial velocities while T was maintained at 250 K. After 5 ns of relaxation, data was

collected for 140 ns (unless n_{ice} reached 1400), after which a second-round of 500 30-ns parallel seeded unbiased MD simulations were performed. In this round, we randomly selected the seeds used as the initial structures from the first round of simulations.

Supplementary Method 2. Ice detection

We used the average bond order parameter[14,15] to characterize the local structures of the water molecules. Specifically, the average bond order parameter calculates the local order of an atom with its neighbors as follows:

$$\bar{q}_l(i) = \left(\frac{4\pi}{2l+1} \sum_{m=-l}^{m=l} \left| \frac{1}{N_i+1} \sum_{j=0}^{N_i} q_{l,m}(j) \right|^2 \right)^{1/2} , \quad (1)$$

where N_i is the number of closest neighbors *j* to atom *i*, and $Y_{l,m}$, $\mathbf{r}_j$, and $\mathbf{r}_k$ in $q_{l,\mathrm{m}}(j) = \frac{1}{N_j} \sum_{k=1}^{N_j} Y_{l,m}(\mathbf{r}_j - \mathbf{r}_k)$ represent the spherical harmonics, position vectors of *j,* and its neighbor *k*, respectively. For bulk water, $\bar{q}_6 = 0.45$ is applied to differentiate hexagonal ice[3] from liquid water when $N_i = 4$. For water at the interface, within 0.45 nm (one layer of water) above the surface, the regions ($\bar{q}_6 > 0.5$, $\bar{q}_4 < 0.6$) and ($\bar{q}_4 > 0.6$) with $N_i = 3$ are employed to characterize hexagonal ice and rhombic ice[3], respectively. Note that the quadrilateral structure with high four-fold symmetry is termed as "square ice" in the reference[3], whereas here we use the term "rhombic ice" since the quadrilaterals are buckled and not always in a perfectly square shape, as shown in Fig. 1a in the main text.

Supplementary Method 3. Construction and validation of MSMs for *T*=230 K

Markov State Models (MSMs) constructed from many simulations provide a promising approach to elucidate the kinetic ensemble of ice nucleation pathways. The basic concept of MSMs is to model the continuous dynamics as Markovian transitions among discrete partitions of configuration space. For ice nucleation, the MSMs first partition the configurational space of interest into a discrete number of states, and then compute the transition probability between pairs of the states at a discretized lag time τ from the parallel MD simulations. If the lag time τ is long enough to allow full relaxation within each state, the fast motions are integrated out and the model

becomes Markovian, i.e., the probability for the system to visit a given state at time $t + \tau$ depends only on its current position at time t. Under this condition, the long-timescale dynamics can be modelled by a sequence of Markovian transitions among the discrete states based on the transition probability.

We constructed MSMs to study the kinetics of heterogeneous ice nucleation (HIN) at T=230 K through the following procedures: (i) From a pool of 18 candidates obtained based on physical intuition (as listed in Supplementary Table 1), we utilized Spectral-oASIS[16,17] to automatically select five collective variables (CVs) that could precisely describe the kinetics of HIN (see Supplementary methods 3.1); (ii) We grouped the MD configurations with the five optimal CVs into 1000 microstates based on the *k*-centers clustering algorithm[18]. The hyperparameter for clustering, i.e., the number of microstates, was determined by conducting variational cross-validation with a generalized matrix Rayleigh quotient (GMRQ)[19] (Supplementary methods 3.2); (iii) We validated our microstate MSMs by implied timescale analysis and the Chapman-Kolmogorov test[20] (see Supplementary methods 3.3); (iv) To better visualize the nucleation kinetics, we further grouped the 1000 microstates into eight macro-states, using the Robust Perron Cluster Clustering Analysis (PCCA+) algorithm[21], as illustrated in Supplementary Fig. 3a. Note that when constructing MSMs, we chose a cut off value (in number of hexagonal ice molecules) when partitioning the configuration space sampled by the MD trajectories into a discrete set of micro-states. As shown in Supplementary Fig. 5 and 6, after the number of hexagonal ice reaches about 800, the system has already entered the stage of continuous ice growth, as indicated by the linear increase in the number of hexagonal ice. Thus, we believe that the MSM with a cut-off at 1200 hexagonal ice molecules is sufficient to analyze the ice nucleation events.

3.1 Selections of representative collective variables

In practice, reducing the feature dimensions by selecting representative CVs that can accurately describe the kinetics of HIN is crucial before constructing the MSMs. Spectral-oASIS[16,17] provides a powerful approach to fulfil this task as it can automatically choose representative CVs that can reconstruct the slowest dynamics relevant to ice nucleation. Starting from a random initial CV, we used Spectral-oASIS to add the CVs one-by-one from the pool of 18 CVs until the time-lagged correlation matrices generated by the selected subset of CVs could well

approximate the leading eigenvalues and eigenvectors of the full matrix of 18 CVs (see Supplementary Table 1). The lag-time was pre-determined as 5 ns, which will be further verified in subsequent Sections. As shown in Fig. 1c in the main text, with the five optimal CVs (i.e., the number of ice molecules in the largest ice nucleus, rhombic ice in the largest ice nucleus, and hexagonal ice in the 2nd, 3rd, and upper layers of the largest ice nucleus), the slowest timescales (which correspond to eigenvalues of the time-lagged correlation matrix) are close to those of the complete 18 CVs, indicating that the reconstructed time-lagged correlation matrix is sufficient to reproduce the slowest dynamical information of the whole set with minimal errors. Furthermore, we have elucidated the contributions of five chosen collective variables (CVs) to the three slowest timescales (see Supplementary Fig. 13) via their relative contributions to three leading eigenvectors from our spectral-oASIS analysis, as shown in Fig. 1c in the main text. Interestingly, we find that the three slowest timescales are strongly correlated with the formation of ice in different layers. For instance, as shown in Supplementary Fig. 13 (top panel), the slowest timescale is mainly related to the CVs that represent the number of rhombic ice molecules in the largest ice nucleus and the total number of ice molecules. These two CVs can well describe the formation of ice in the first layer in contact with the surface, as rhombic ice mainly forms in the first layer and the number of hexagonal ice molecules can be obtained by deducting the number of rhombic ice molecules from the number of total ice molecules. Consistently, the formation of ice patches in the first layer indeed corresponds to the rate limiting step (the slowest timescale process) in both of our classical (formation of purely hexagonal ice mainly in the first layer) and non-classical ice nucleation pathways (formation of the mixture of rhombic ice and hexagonal ice mainly in the first layer). The second-slowest timescale and third-slowest timescale are mainly related to the formation of hexagonal ice in the third layer and second layer of the largest ice nucleus, respectively. Notably, the timescale for the formation of hexagonal ice in the third layer (middle panel, Supplementary Fig. 13) is found to be slower than that in the second layer (bottom panel, Supplementary Fig. 13). This observation underlines the second step in our two-step non-classical nucleation pathway, where the rhombic ice needs to be converted into hexagonal ice. This conversion process mainly involves the first two layers of ice, as the transformation of the rhombic ice molecule in the first layer will free one of its -OH bonds to quickly form a hydrogen bond with a hexagonal ice molecule in the 2nd layer. As a result, the growth of the 3rd layer of hexagonal ice

is contingent upon the completion of this conversion process, rendering it to be the second-slowest timescale.

3.2 Determination of hyperparameters and clustering

We first determined an appropriate number of microstates by conducting variational cross-validation with GMRQ[19]. In the cross-validation, the MD trajectories were randomly divided into five folds, in which four folds were used as a training set, and one fold was used as a testing set. The optimal number of microstates was determined to be 1000 since it had the highest test score before entering the overfitting regime, as indicated by the progressively decreasing score shown in Supplementary Fig. 14. Then, the MD configurations were grouped into 1000 microstates based on the *k*-centers clustering algorithm[18]. The GMRQ cross-validation and clustering were performed by using in-house python codes in MSMbuilder Packages[22].

3.3 Validations of MSMs

We constructed our microstate MSMs using a lag-time of 5 ns, as the implied timescale plateaued at this lag-time (see Supplementary Fig. 3b), indicating that our microstate MSMs reach Markovian. To further verify our MSMs, we conducted Chapman-Kolmogorov test[20] by comparing the residence probabilities of the ten most populated microstates predicted by our MSMs with those directly counted from original MD data. This comparison showed that our MSMs are consistent with the original MD simulations when predicting the dynamics, as displayed in Supplementary Fig. 4.

Supplementary Method 4. Construction and validation of MSMs at higher temperatures

We also constructed MSMs at T=240 K and 250 K. Before the MSM construction, we have also conducted spectral-oASIS analysis at T=240 K and 250 K. As shown in Supplementary Fig. 15, we also need around five CVs to reproduce the slowest timescales of the completed sets of CVs. Furthermore, we found that the selected CVs is largely overlapped with those selected at 230K. For instance, in both T=240 K and 250 K, the five chosen CVs by Spectral-oASIS are: the number of ice molecules in the largest ice nucleus, number of hexagonal ice in the 2nd, 3rd, and upper layers of the largest ice nucleus, and the number of ice molecules in the largest hexagonal ice nucleus. The most notable difference is that the CV that represents the number of rhombic ice

is no longer selected at 240K and 250K. This observation agrees well with one of our key conclusions that the non-classical two-step pathway (where the rate-limiting step involves the formation of rhombic ice) becomes less significant as temperature increases. In order to compare the flux of the classical and non-classical pathways at different temperatures, we adopted the same CVs and state-definition obtained at T=230K across all three temperatures. In addition, we have also performed a control analysis to show that these five CVs chosen at 230K (largely overlapped with CVs chosen by Spectral-oASIS at 240K and 250K) can still reasonably reproduce the slowest timescales at these two higher temperatures (see right panels in Supplementary Fig. 15a and b for 240K and 250K, respectively). Note that extra care should be taken since some of the microstates are missing at higher temperatures. The microstate MSMs were also validated by implied timescale analysis and the Chapman-Kolmogorov test. Supplementary Fig. 9b and 10b show that the implied timescales plateau for both cases at t=4 ns and 1.5 ns, respectively, implying that the MSMs reach Markovian. In addition, the residence probabilities for the most populated microstates in Supplementary Fig. 9c and 10c further validate the MSMs for T=240 K and 250 K, respectively.

Supplementary Method 5. Obtaining long-time trajectories and nucleation rates by Markov Chain Monte Carlo Sampling based on microstate MSM

When the probability of the system taking any of its 1000 microstates at time t is denoted as vector $\mathbf{p}(t)$, the probabilities at time $t + \tau$ can be given by $\mathbf{p}(t + \tau) = \mathbf{p}(t)T(\tau)$, where $T(\tau)$ is the transition probability matrix with a lag time of τ. Thus, with the transition probability matrix obtained from microstate MSMs, Markov Chain Monte Carlo simulations can be performed to simulate the Markov jumps based on the function of $\mathbf{p}(t + \tau) = \mathbf{p}(t)T(\tau)$ to correctly generate the long-time trajectories.

From the generated simulations, we can obtain the mean first passage times (MFPTs) by calculating the average transition times between pairs of states of interest. Subsequently, the nucleation rate J can be estimated by $J = \frac{1}{t_{\mathrm{MFPT}} V}$, where t_{MFPT} is the MFPT for the system to enter the growth stage, and V is the volume of water in the system[23,24]. Specifically, $t_{\mathrm{MFPT}} = \frac{\sum_i p_i t^i_{\mathrm{MFPT}}}{\sum_i p_i}$, where p_i is the percentage of flux i, and t^i_{MFPT} is the MFPT for the system from macro-state I to

macro-states IV or V for flux i. Note that the heterogeneous nucleation rate should normally be evaluated using the surface area (for nucleation on a surface). Here, a volume-based nucleation rate is used to allow for a direct comparison with homogeneous nucleation[23].

Supplementary Method 6. Identification of the critical ice nucleus via committor probability analysis

The flux of the nucleation pathways was analysed based on a mathematical framework of TPT[25–28]. We computed the flux of ensembled nucleation pathways along with their relative probabilities at the microstate level. For visualization purposes, the net flux of the transitions was assembled into coarse-grained macrostates[29]. To identify the critical ice nuclei along the classical and non-classical pathways, the saddle points along the two pathways were obtained using two independent TPT analyses based on the committor analysis at the microstate level. As the rate-limiting step of the nucleation flux is proximal to where the flux bifurcates, the committor analyses were located around the bifurcation. For TS I and TS II, the transition microstates can be identified with a corresponding committor probability of 0.5, meaning they have a 50% probability to return to state I, while the other 50% corresponds to the probability to proceed to the microstates in state IV and III, respectively (see Fig. 3a in the main text).

Supplementary References

1. Frenkel, D. & Smit, B. *Understanding molecular simulation* (Elsevier, 2002). doi:10.1016/b978-0-12-267351-1.x5000-7
2. Plimpton, S. Fast parallel algorithms for short-range molecular dynamics. *J. Comput. Phys.* **117**, 1–19 (1995).
3. Li, C., Gao, X. & Li, Z. Surface energy-mediated multistep pathways for heterogeneous ice nucleation. *J. Phys. Chem. C* **122**, 9474–9479 (2018).
4. Abascal, J. L. F., Sanz, E., Fernández, R. G. & Vega, C. A potential model for the study of ices and amorphous water: TIP4P/Ice. *J. Chem. Phys.* **122**, 234511 (2005).
5. Ryckaert, J. P., Ciccotti, G. & Berendsen, H. J. C. Numerical integration of the cartesian equations of motion of a system with constraints: molecular dynamics of n-alkanes. *J. Comput. Phys.* **23**, 327–341 (1977).
6. Andersen, H. C. Rattle: A 'velocity' version of the shake algorithm for molecular dynamics calculations. *J. Comput. Phys.* **52**, 24–34 (1983).
7. Jorgensen, W. L., Chandrasekhar, J., Madura, J. D., Impey, R. W. & Klein, M. L. Comparison of simple potential functions for simulating liquid water. *J. Chem. Phys.* **79**, 926–935 (1983).
8. Lu, G., Hu, H., Duan, Y. & Sun, Y. Wetting kinetics of water nano-droplet containing non-surfactant nanoparticles: A molecular dynamics study. *Appl. Phys. Lett.* **103**, 253104 (2013).
9. Hockney, R. W. & Eastwood, J. W. *Computer simulation using particles*. (CRC Press, 1988).
10. Parrinello, M. & Rahman, A. Polymorphic transitions in single crystals: A new molecular dynamics method. *J. Appl. Phys.* **52**, 7182–7190 (1981).
11. Martyna, G. J., Tobias, D. J. & Klein, M. L. Constant pressure molecular dynamics algorithms. *J. Chem. Phys.* **101**, 4177–4189 (1994).
12. Shinoda, W., Shiga, M. & Mikami, M. Rapid estimation of elastic constants by molecular dynamics simulation under constant stress. *Phys. Rev. B* **69**, 134103 (2004).
13. Tuckerman, M. E., Alejandre, J., López-Rendón, R., Jochim, A. L. & Martyna, G. J. A Liouville-operator derived measure-preserving integrator for molecular dynamics simulations in the isothermal-isobaric ensemble. *J. Phys. A. Math. Gen.* **39**, 5629–5651 (2006).
14. Steinhardt, P. J., Nelson, D. R. & Ronchetti, M. Bond-orientational order in liquids and glasses. *Phys. Rev. B* **28**, 784–805 (1983).
15. Lechner, W. & Dellago, C. Accurate determination of crystal structures based on averaged local bond order parameters. *J. Chem. Phys.* **129**, 114707 (2008).
16. Drineas, P. & Mahoney, M. W. On the nyström method for approximating a gram matrix for improved kernel-based learning. *J. Mach. Learn. Res.* **6**, 2153–2175 (2005).
17. Litzinger, F. *et al.* Rapid calculation of molecular kinetics using compressed sensing. *J. Chem. Theory Comput.* **14**, 2771–2783 (2018).
18. Bowman, G. R., Beauchamp, K. A., Boxer, G. & Pande, V. S. Progress and challenges in the automated construction of Markov state models for full protein systems. *J. Chem. Phys.* **131**, 124101 (2009).
19. McGibbon, R. T. & Pande, V. S. Variational cross-validation of slow dynamical modes in molecular kinetics. *J. Chem. Phys.* **142**, 124105 (2015).

20. Prinz, J. H. *et al.* Markov models of molecular kinetics: Generation and validation. *J. Chem. Phys.* **134**, 174105 (2011).
21. Deuflhard, P. & Weber, M. Robust Perron cluster analysis in conformation dynamics. *Linear Algebra Appl.* **398**, 161–184 (2005).
22. Harrigan, M. P. *et al.* MSMBuilder: Statistical models for biomolecular dynamics. *Biophys. J.* **112**, 10–15 (2017).
23. Bi, Y., Cao, B. & Li, T. Enhanced heterogeneous ice nucleation by special surface geometry. *Nat. Commun.* **8**, 15372 (2017).
24. Li, C. *et al.* Enhancing and impeding heterogeneous ice nucleation through nanogrooves. *J. Phys. Chem. C* **122**, 25992–25998 (2018).
25. E, W. & Vanden-Eijnden, E. Towards a theory of transition paths. *J. Stat. Phys.* **123**, 503–523 (2006).
26. Metzner, P., Schütte, C. & Vanden-Eijnden, E. Transition path theory for markov jump processes. *Multiscale Model. Simul.* **7**, 1192–1219 (2009).
27. E, W. & Vanden-Eijnden, E. Transition-path theory and path-finding algorithms for the study of rare events. *Annu. Rev. Phys. Chem.* **61**, 391–420 (2010).
28. Berezhkovskii, A., Hummer, G. & Szabo, A. Reactive flux and folding pathways in network models of coarse-grained protein dynamics. *J. Chem. Phys.* **130**, 205102 (2009).
29. Noé, F., Schütte, C., Vanden-Eijnden, E., Reich, L. & Weikl, T. R. Constructing the equilibrium ensemble of folding pathways from short off-equilibrium simulations. *Proc. Natl. Acad. Sci. U. S. A.* **106**, 19011–19016 (2009).
30. Zeng, X. *et al.* Elucidating dominant pathways of the nano-particle self-assembly process. *Phys. Chem. Chem. Phys.* **18**, 23494–23499 (2016).
